# At what age endometriosis-associated ovarian cancer is diagnosed? The implications for women in the reproductive age

**DOI:** 10.3389/fonc.2023.1193123

**Published:** 2023-05-23

**Authors:** Johnny S. Younis, Ido Izhaki

**Affiliations:** ^1^ Reproductive Medicine, Department of Obstetrics and Gynecology, Baruch-Padeh Medical Center, Poriya, Israel; ^2^ Azrieili Faculty of Medicine, Bar-Ilan University, Safed, Israel; ^3^ Department of Evolutionary and Environmental Biology, University of Haifa, Haifa, Israel

**Keywords:** endometriosis, endometrioma, endometriosis-associated ovarian cancer, clear-cell ovarian carcinoma, endometrioid ovarian cancer, reproductive age, age at diagnosis.

## Introduction

Endometriosis-associated ovarian cancer (EAOC) is an evolving distinct clinical entity and challenge considered to develop from endometrioma (1). Several systematic reviews and meta-analyses corroborated the association between endometriosis and ovarian cancer (2–5). In the most recent and comprehensive, the summary relative risk of EAOC was estimated as 1.93 compared to women with no endometriosis (6). Furthermore, contemporary state-of-the-art methodologies have provided evidence of genetic correlation and causal relationship between endometriosis and EAOC (7).

Clear-cell and endometrioid ovarian epithelial carcinomas are the most intensely and reproducibly associated malignancies with endometriosis. While endometriosis may also be associated with low-grade serous ovarian carcinoma, this linkage is not well documented (8). Coexistence with endometriosis is observed in about 20%–50% of all women with clear-cell and endometrioid ovarian carcinomas (9–11). The increased risk of developing clear-cell and endometrioid ovarian carcinomas in women with endometriosis is 3.4 and 2.3-fold, respectively (6).

## The rationale for the current opinion

Endometriosis is a widespread, chronic, inflammatory, and estrogen-dependent condition, and endometrioma is the most pathognomonic and diagnosed form of the disease. Their estimated prevalence is 1 in 10 and 1 in 18, respectively, in women of reproductive age (12). Therefore, the diagnosis of EAOC in women with endometriosis, particularly with an intact endometrioma, may raise much concern. Furthermore, the link and its translation into clinical practice regarding information to patients and early cancer detection still need to be clarified (13).

Overall, ovarian cancer is a postmenopausal disease, although, in about 12% of cases, it may develop in women < 44 years of age (14). This estimate includes numerous women with borderline and non-epithelial tumors, typically presenting at a young age (15, 16). Overall, women with EAOC are older than those with benign endometrioma but younger than women with non-EAOC ovarian carcinomas, such as high-grade serous ovarian carcinoma. Additionally, although there are several case reports of EAOC at a young age (17–20), the exact age distribution of EAOC diagnosis is still not well-expounded.

Furthermore, although age was suggested as a risk factor in cases with EAOC, many discrepancies exist between reports (21–24). For example, previous publications reported a wide range of EAOC premenopausal diagnosis rates between 30 and 70% of cases (21, 25–28). Moreover, due to the disease’s infrequency, publications exploring EAOC in the last decade were retrospective in design and included a modest number of women, the largest including 163 women (29).

Collectively, the age at EAOC diagnosis is still not well delineated. Although endometrioma is a prevalent manifestation in the reproductive age, EAOC may seem exceptional. A methodical assessment of age at EAOC diagnosis may have numerous implications on the clinical management of women with intact endometrioma, especially in planning future pregnancies. It may clarify the chance of EAOC diagnosis stratified by age and direct physicians on advising, following, and treating their patients relying on relevant medical evidence. Moreover, it may supplement essential information to reproductive endocrinologists and gynecological surgeons in their counseling for the best treatment approach, especially when atypical features of an endometrioma appear on transvaginal ultrasound (TVUS).

## Methods

To reach the objective of this opinion paper, a search for cohort studies published in the English literature was performed on Pubmed.com from January 2011 to October 2022, addressing EAOC, clear-cell ovarian cancer, and endometrioid ovarian cancer. Cohort studies that targeted women with EAOC elaborating on the mean age ± standard deviation (SD) or median and interquartile range (IQR) or range (minimum and maximum) were included in the quantitative assessment. The diagnosis of EAOC should have been performed surgically and approved by pathology.

The keywords ‘endometriosis’, ‘endometrioma’, ‘ovarian endometriosis’, ‘endometriosis-associated ovarian cancer’, ‘clear-cell ovarian carcinoma’, ‘endometrioid ovarian carcinoma’, ‘age’, and ‘reproductive age’ were included. The relevance of reached publications was evaluated following reading the abstract. Case reports and reviews were excluded. Furthermore, publications that assessed only a specific age group of women were omitted from the evaluation. Articles with an inappropriate design were also excluded.

## Statistical evaluation

We assumed normal distribution for the statistical analyses of women’s age since this information was not specified in the 25 studies included in our analysis. The mean and standard deviation of women’s age were reported in 17 studies. For the other studies where only median and IQR or median and minimum/maximum were given, mean and SD were approximated using the method of Wan et al. (30). The weighted mean and SD were calculated with the number of participants in each study as the weighting factor, using the formulas of N.I.S.T (31) for each of the four studied groups (all eligible studies combined, studies that targeted EAOC originating from ovarian endometriosis, studies that were conducted in countries with a high incidence of EAOC and studies that included women solely with clear-cell ovarian carcinoma). Using z-scores, we calculated the percentage of women in the four groups below 50, 45, 40, and 35 years.

## Results

Twenty-five studies (27 cohorts) were eligible for the quantitative age evaluation at EAOC diagnosis, including 1082 cases (**Table 1**). The diagnosis of EAOC was performed surgically and examined by experienced pathologists in all studies (21–25, 27–29, 32–48).

**Table 1:** Age at diagnosis of endometriosis-associated ovarian cancer – a summary of cohort studies.

Study	No. of women	Country	Study methodology	Study period (years)	Age (years) Mean +/- SD	Age range (years)	Notes	Cancer arising from ovarian endometriosis
Hernández et al., 2022 (23)	17	Spain	Retrospective observational	3	Median 50	Interquartile 43-63	All with EAOC	Yes
Huang et al., 2022 (24)	57	Taiwan	Retrospective cooperative	3	53.1 ± 9.3	ND	All with CCOC 47/57 cases associated with endometriosis	Yes
So et al., 2021 (44)	12	South Korea	Retrospective	17	36.3 ± 5.2	29-56	All with EAOC	Yes
Zhang et al., 2021 (47)	14	China	Retrospective comparative	9	47.1 ± 11.0	ND	All with EAOC	Yes
Zhou et al., 2021 (46)	114	China	Retrospective	21	51.1 ± 9.0	24–79	All with EAOC	ND
Zhu et al., 2021 (45)	16	China	Retrospective	10	45.5 ± 6.2	35-54	All with CCOC associated with endometriosis	Yes
Udomsinkul et al., 2020 (20)	79	Thailand	Retrospective case-control	16	Median 49	Interquartile 44-54	All with EAOC	Yes
Li et al., 2019 (25)	34	China	Retrospective comparative	17	48.7 ± 9.0	32-63	All with EAOC	Yes
Son et al., 2019 (44)	35	South Korea	Retrospective comparative	17	Median 47	33-60	All with CCOC associated with endometriosis	Yes
Bassiouny et al., 2018 (29)	168	Canada	Retrospective comparative	13	56.6 ± 11.5	ND	All with EAOC	Yes
Yamamoto et al., 2018 (43)	28	Japan	Retrospective comparative	4	53.1 ± 14.5	ND	All with EAOC	Yes
Moro et al., 2018 (27)	49	European countries	Retrospective multicenter	17	Median 53	26-86	All with EOC associated with endometriosis	Yes
Muangtan et al., 2018 (42)	32	Thailand	Retrospective comparative	5	51.4 ± 10.1	ND	All with EAOC	Yes
Pozzati et al., 2018 (28)	24 10 20	European countries	Retrospective multicenter	17	Median 47.5 Median 55.0 Median 55.0	32-72 37-80 35-70	All with CCOC associated with endometriosis	Yes Yes No
Paik et al., 2018 (41)	41	South Korea	Retrospective comparative	15	45.1 ± 7.0	ND	All with EAOC	Yes
Park et al., 2018 (40)	78	South Korea	Retrospective comparative	22	Median 48	29-69	All with CCOC associated with endometriosis	Yes
Tanase et al., 2018 (39)	40	Japan	Retrospective comparative	8	54.3 ± 8.9	39-75	All with EAOC	Yes
Kuo et al., 2017 (38)	11	Thailand	Retrospective	10	44.7 ± 3.2	40-52	All with EAOC	Yes
Bounus et al., 2016	45	Italy	Retrospective comparative	10	59.0 ± 9.6	ND	All with EAOC	No 34/45 of cases had extra-gonadal endometriosis
Dinkelspiel et al., 2016 (36)	49	USA	Retrospective comparative	14	Mean 52 Median 52	47-57	All with EAOC	Yes
Acién et al., 2015 (35)	20	Spain	Retrospective comparative	20	48.8 ± 11.6	32-72	All with EAOC	No
Akbarzadeh-Jahromi et al., 2015 (34)	28	Iran	Retrospective multi-center	6	49.9± 9.4	29-72	All with EAOC	No Included cases with extra-gonadal endometriosis
Scarfone et al., 2014 (33)	27	Italy	Retrospective comparative	22	51.4 ± 10.0	30-71	All with CCOC associated with endometriosis	Yes
Wang et al., 2013 (21)	17	China	Retrospective comparative	1	46.1 ± 10.1	33-66	All with EAOC	No Included cases with extra-gonadal endometriosis
Kondi-Pafiti et al., 2012	17	Greece	Retrospective	10	Median 58	26-76	All with EAOC	Yes

ND - not disclosed; EAOC - endometriosis-associated ovarian cancer; CCOC - clear-cell ovarian cancer; EOC - endometrioid ovarian cancer.

Eleven studies were excluded from the quantitative evaluation. Five studies did not disclose EAOC patients’ age, SD, or range (49–53). Two studies assessed only EAOC women above 45 or below 40 years (26, 54). One study targeted women following endometrioma resection (55), and one more study evaluated only EAOC cases resistant to platinum (56). Other studies did not meet the inclusion criteria (57, 58).

The characteristics of eligible 25 studies are précised in **Table 1**. All studies were retrospective in design. The studies originated from countries around the globe in Asia, Europe, and North America. Most studies (19/25) summarized an extended institutional clinical experience of more than seven years. In all studies, the mean ( ± SD) duration invested in EAOC institutional evaluation was 12.26 ± 6.39 years.

Eighteen studies assessed all cases of EAOC combined (21–23, 25, 29, 32, 34–39, 41–43, 46–48), six evaluated only patients with clear-cell ovarian cancer associated with endometriosis (24, 28, 33, 40, 44, 45), and one study endometrioid ovarian cancer associated with endometriosis (27). In addition, six eligible studies were performed in countries with a higher prevalence of EAOC, in Japan, Thailand, and Taiwan (22, 24, 38, 39, 42, 43). Nineteen eligible studies included EAOC cases arising within the endometrioma following Sampson and Scott’s criteria (59, 60). In contrast, six studies included patients with extra-gonadal endometriosis (61), or the subtype of endometriosis was not disclosed (**Table 1**).

Assessing all eligible studies conjointly, the mean age of the 1082 women at EAOC diagnosis was 51.64 ± 3.24 years. Among these women, 30.68% and 2.10% were below 50 and 45 years, respectively, equivalent to 31:100 and 2:100 among all women assessed. On the other hand, EAOC diagnosis in women below 40 and 35 years resulted in much lower estimates corresponding to 0.017% and <0.001%, equivalent to 2:10,000 and <1:100,000, respectively.

Nineteen studies targeted EAOC originating from ovarian endometriosis and included 838 cases with a mean age of 51.51 ± 3.29 years. Among these women, 32.26% and 2.38% were below 50 and 45 years of age, equivalent to 32:100 and 2:100, respectively. However, EAOC diagnosis in this group of women below 40 and 35 years resulted in much lower estimates corresponding to 0.023% and <0.001%, equivalent to 2:10,000 and <1:100,000, respectively.

Six studies were conducted in countries with a high incidence of EAOC and included 247 cases with a mean age of 51.39 ± 3.30 years. Of these women, 33.71%, 2.66%, 0.028%, and <0.001% were below 50, 45, 40, and 35 years, equivalent to 34:100, 3:100, 3:10,000, and <1:100,000, respectively.

Six studies included women solely with clear-cell ovarian carcinoma and involved 267 cases with a mean age of 50.27 ± 3.58 years. Of these women, 46.95%, 7.02%, 0.20%, and 0.001% were below 50, 45, 40, and 35 years, equivalent to 47:100, 7:100, 2:1000, and 1:100,000, respectively.

## Discussion

Our methodical assessment, summarizing 25 carefully chosen studies (27 cohorts) and including 1082 EAOC cases, shows that EAOC diagnosis is a disease of the menopausal age. These global results rely on an extended experience of 12.26 ± 6.39 years invested among various institutional practices. In our analysis of all eligible studies, the mean age of women with EAOC was 51.64 ± 3.24 years. Nonetheless, 31% of women were < 50 years of age, most probably peri- or premenopausal, and only 2% of patients were <45 years of age, apparently diagnosed during the reproductive period. Furthermore, the diagnosis of EAOC in young women <40 and <35 years of age was sporadic, equivalent to 1:5,000 and <1:100,000, respectively. These estimates did not change when EAOC cases in women with extra-gonadal endometriosis were excluded from the evaluation.

Estimates of premenopausal EAOC diagnosis in countries with a higher disease prevalence, Japan, Thailand, and Taiwan, seem to increase slightly. Almost 34:100, and 3:100, of women developing EAOC in those countries, were <50 and <45 years, respectively. However, only 3:10,000 and <1:100,000 were below <40 and <35 years, respectively.

Furthermore, studies that evaluated only clear-cell ovarian cancer associated with endometriosis showed a further increase in premenopausal diagnosis. Almost 47:100 and 7:100 of women with this type of cancer were diagnosed <50 and <45 years, respectively. However, no significant clinical change in this group was diagnosed in young women <40 and <35 years. Therefore, it is plausible that the slight increase in the premenopausal diagnosis of EAOC in Japan, Thailand, and Taiwan is caused by the predominance of clear-cell ovarian carcinoma in these countries (62).

Taken together, our results demonstrate that a malignant transformation of a benign endometrioma to EAOC in the reproductive age is a sporadic event, notably in women <40 years of age. In women of late reproductive age, between 40-45 years, EAOC is diagnosed in almost 2-3% of cases. In cases with clear-cell ovarian carcinoma associated with endometriosis, our results imply that up to 7% of cases are diagnosed in this age group. These estimates are clinically significant since endometriosis and endometrioma are prevalent at young ages, estimated at 1 in 10 and 1 in 18, respectively. They may be beneficial to ease the concerns of these patients and their attending practitioners. Furthermore, these estimates may be employed for proper guidance and counseling of women of reproductive age with an intact endometrioma planning for a future pregnancy.

Our results, assessing a total of 1082 cases, demonstrates the age diversity at diagnosis between patients with EAOC and non-EAOC, specifically high-grade serous ovarian cancer (HGSOC). The mean age of women with HGSOC, the most common and lethal type of ovarian cancer, is 63 years (63), almost 12 years older than EAOC.

Furthermore, our analyses substantiate previous studies, counting on modest numbers, indicating that age is an independent risk factor in cases of EAOC (21–24); and translating these risks into informatics that may be introduced into the clinical setting. Our calculations also imply that EAOC should not be an argument for surgical treatment of an intact endometrioma in the reproductive age, particularly in cases planning a future pregnancy. It is well recognized today that endometriotic cystectomy significantly reduces ovarian reserve (64, 65), estimated by 39% and 57%, in uni- and bilateral cases, 9-12 months following surgery, suggesting a long-standing impact on women’s reproductive life span (65).

Although EAOC diagnosis at the reproductive is infrequent and even sporadic in women <40 years, the index of suspicion should be directed into distinct clinical situations. Cases with relapsing or worsening pelvic pain, the rapid growth of an endometrioma, or alternately larger-sized endometrioma, particularly >9 cm, should be perused to investigate EAOC (12). In this setting, serum cancer antigen 125 has no added benefit (66, 67).

Clinically, imaging is vital in evaluating and differential diagnosing an endometrioma transformation to an EAOC, noticeably in reproductive age. Pelvic transvaginal ultrasound (TVUS) is the first-line imaging mode, while computerized tomography scan performs poorly. Magnetic resonance imaging (MRI) is a valuable adjunct for ovarian findings described as intermediate or atypical by TVUS (68, 69).

On TVUS, benign endometrioma appears as an ovarian mass with a homogenous ‘ground glass’ appearance, uni- or bilateral, without solid parts or papillations. However, as age increases, papillations and other solid parts become more frequent, and the ‘ground glass’ appearance becomes less common, while cyst diameter seems to stay the same (70). Conversely, EAOC, specifically clear-cell and endometrioid ovarian carcinomas, are large, above 9 cm, with a mean size of 11-13 cm, unilateral tumors with solid components, papillary projections, and vascularization (12, 71).

In clinical practice, about 5–25% of cases will have indeterminate or atypical adnexal findings by TVUS (72). Since most of these cases may be benign, MRI performance is crucial in managing and counseling these cases. In addition, MRI-supportive performance in such situations may reduce patient anxiety, repeat imaging, unnecessary follow-up, and avoid surgery. On MRI, benign endometriomas typically display features of T2-weighted image shading (72). A larger cyst and an enhanced solid portion of the endometrioma may suggest a malignant transformation (73). Shading disappearance within the endometrioma on T2-weighted images may also mean malignant transformation (39).

Endometrioma and deep infiltrating endometriosis (DIE) occasionally co-exist in the same women (74, 75). In a recent cross-sectional study of 1,191 women with subfertility aged 25–39 years, undergoing a systematic TVUS evaluation, both disease subtypes were found in almost 22% of women (75). Nevertheless, the available evidence supports EAOC rise mainly from endometrioma (1). Malignant transformation of extra-gonadal sites, as in superficial or deep endometriosis, is most unusual (76–78). In a recent narrative review of cases with DIE malignant transformation, only eight patients were collected in ten years (78). Thus, a high level of suspicion should be invested in DIE cases presenting with new clinical manifestations and appearing with a pelvic mass such as on the pelvic wall, pouch of Douglass, and recto-vaginal septum (78). Further studies are essential to explore the association between extra-gonadal endometriosis and malignant transformation.

**Figure 1:**
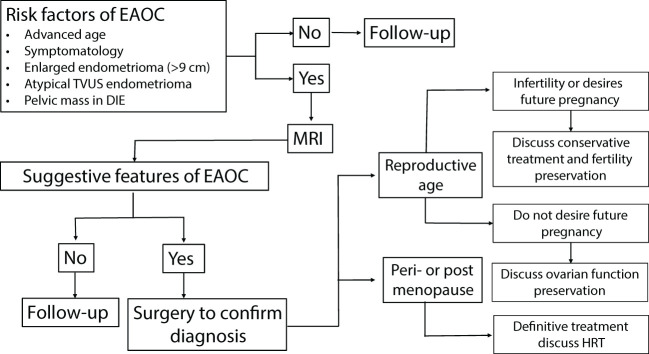
Clinical management of women with an isolated endometrioma or joint with deep infiltrating endometriosis, suspected to have EAOC, in the reproductive age and menopausal transition. EAOC, endometriosis-associated ovarian cancer; TVUS, transvaginal ultrasound; MRI, magnetic resonance imaging; HRT, hormone replacement therapy.

**Figure 1** summarizes in a flow chart our suggested way of clinical management of endometrioma (isolated or joined by DIE) developing manifestations of EAOC, including atypical TVUS endometrioma features. The risk factors for EAOC have been discussed earlier (12) and are summarized in **Figure 1**. MRI performance is mandatory in these cases, especially when atypical endometrioma features appear on TVUS. A definite diagnosis of EAOC necessitates surgery, most commonly by endoscopy. Definitive management should consider the patient’s age, the final pathological diagnosis, stage, and grade of disease. Since women with EAOC are diagnosed at an earlier stage and have a more favorable histological grade (12), conservative surgical management should be discussed in infertile cases or women of reproductive age desiring a future pregnancy.

The time and intervals of follow-up in cases with isolated endometrioma, with typical or atypical TVUS features, is an intricate but essential demand and need more targeted studies to be addressed. Likewise, follow-up of cases with atypical TVUS features and reassuring MRI results need further investigation. Meanwhile, we recommend a multidisciplinary case-by-case consultation (adjoining a gynecological oncologist and reproductive medicine experts) contemplating, age, desire for future pregnancy, symptomatology, ovarian reserve, physical and TVUS findings, and MRI features.

In summary, this is the first report in the literature delineating and expounding the age at EAOC diagnosis, substantiating that age is an independent risk factor for the disease. Our results analyzing 1082 cases from 25 studies demonstrate that EAOC is a menopausal disease, with a mean age of 51.64 ± 3.24 years at diagnosis. About 30.68% of patients with EAOC are <50 years upon diagnosis, presumably premenopausal, equivalent to 31 in 100 women. However, only 2.1%, 0.017%, and <0.001%, apparently during the reproductive age, comparable to 1 in 50, 1 in 5,000, and < 1 in 10,000, are <45, <40, and <35 years, respectively. In cases with clear-cell ovarian carcinoma associated with endometriosis, 47 in 100 and 7 in 100 are below 50 and 45 years, with no clinically significant changes in women <40 and <35 years. Since endometrioma is widespread in women of reproductive age, these estimates are essential for guidance and counseling, especially in women planning for a future pregnancy. Our results imply that the likelihood of EAOC development during reproductive age is reassuring, and this should be translated into clinical practice regarding information to patients. At the same time, a high index of suspicion should remain in special situations when clinical, TVUS, and MRI features suggest EAOC. Conversely, an extended follow-up should be considered in women with large or atypical endometriomas not retracting following menopause.

## Author contributions

JY conceived the idea of this perspective, contributed to study design and data extraction, performed the analyses and data interpretation, and drafted the manuscript; II contributed to study design and execution, contributed to analyses and interpretation of data, performed the statistical analysis, and revised the manuscript for important intellectual content. All authors contributed to the article and approved the submitted version.
